# Supplementary material for: Facile Fabrication of Superwetting PVDF Membrane for Highly Efficient Oil/Water Separation
Source: Polymers (Basel). 2023 Jan 9;15(2):327. doi: 10.3390/polym15020327 (PMC9865060; doi:10.3390/polym15020327)
Supplement: Supplementary file 1 [file polymers-15-00327-s001.zip › polymers-2116986-supplementary.pdf]

polymers
MDPI

Supplementary Materials

Facial Strategy for Preparation of Modified PVDF Membrane: Endowing Effective Separation of Oil/Water Mixtures

Jinzhu Yang, Wei Sun, Junping Ju *, Yeqiang Tan and Hua Yuan *

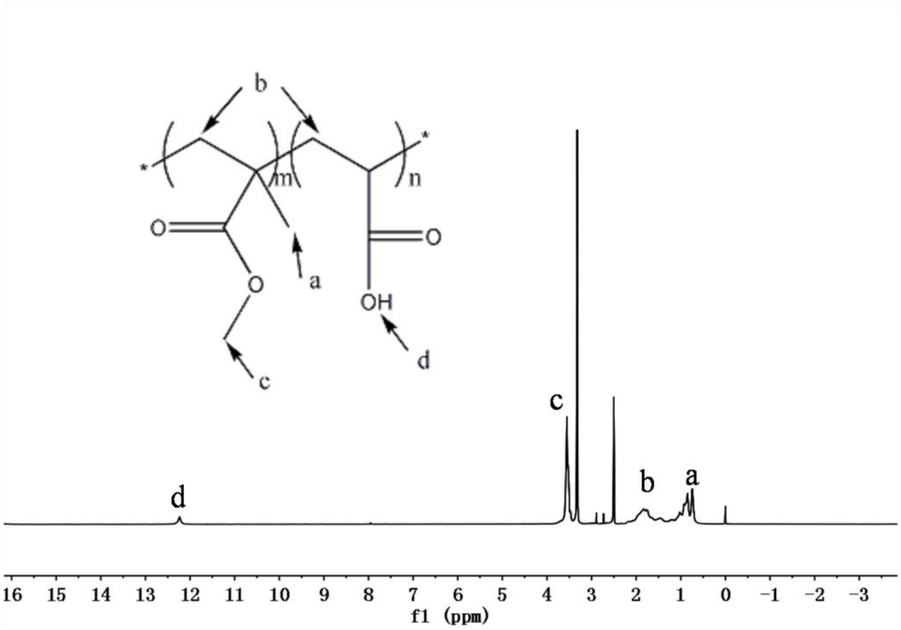

Figure S1. ^{1}H NMR of P (MMA -AA) in d_6-DMSO.

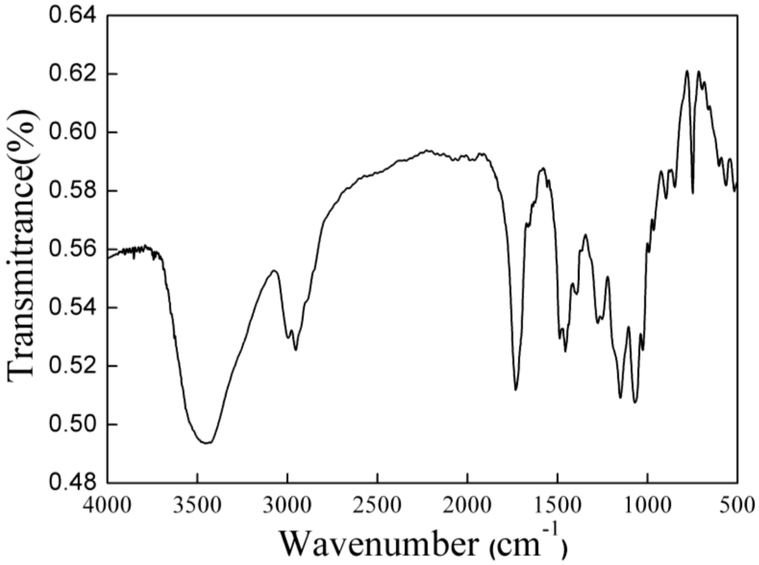

Figure S2. FTIR spectrum of the copolymer P (MMA-AA).

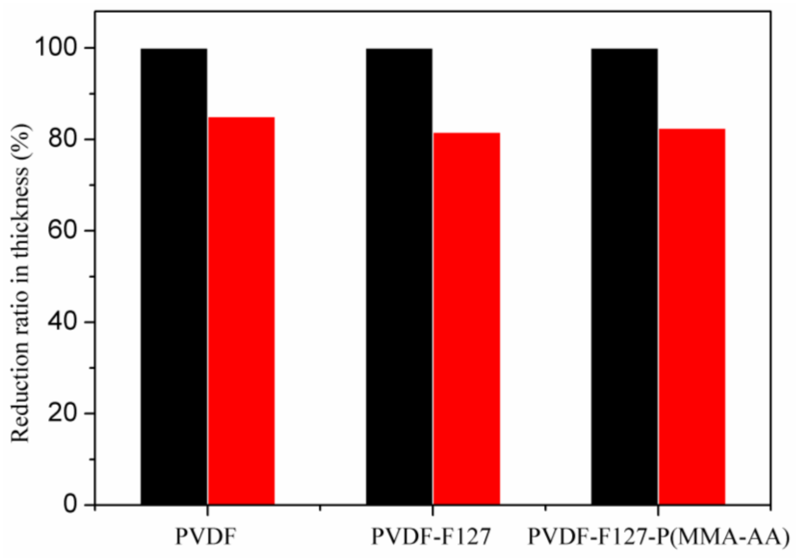

Figure S3. the thickness changes of PVDF, PVDF-F127, PVDF-F127-P (MMA-AA) membranes via heating treatment. (membrane before heating treatment, membrane before heating treatment).

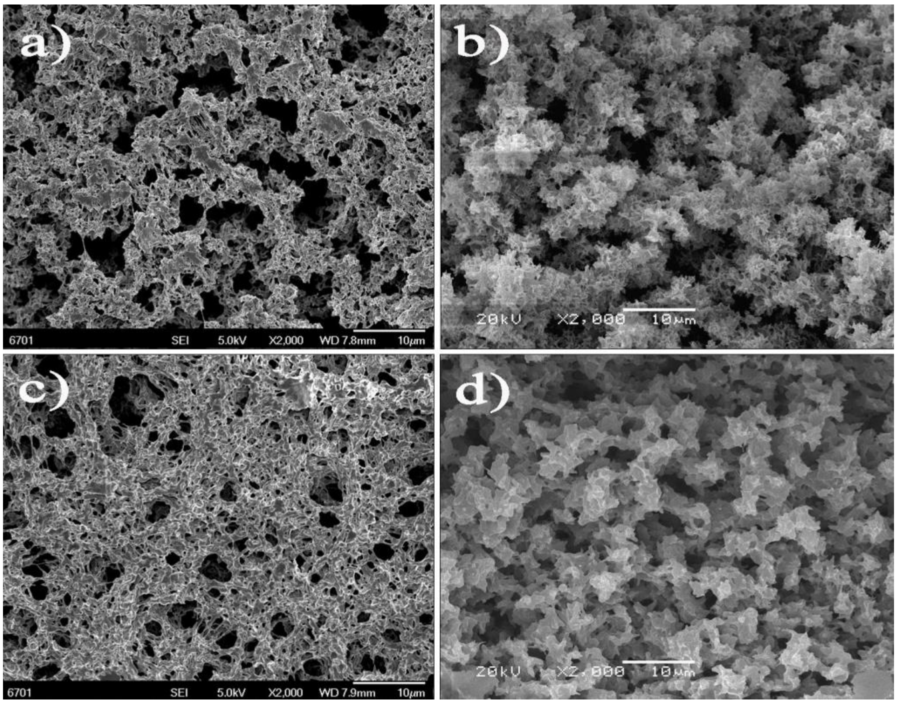

Figure S4. (a,b) surface and cross sectional structure images of the PVDF-F127 membrane. (c,d) surface and cross sectional structure images of the PVDF-F127 membrane after heating treatment.

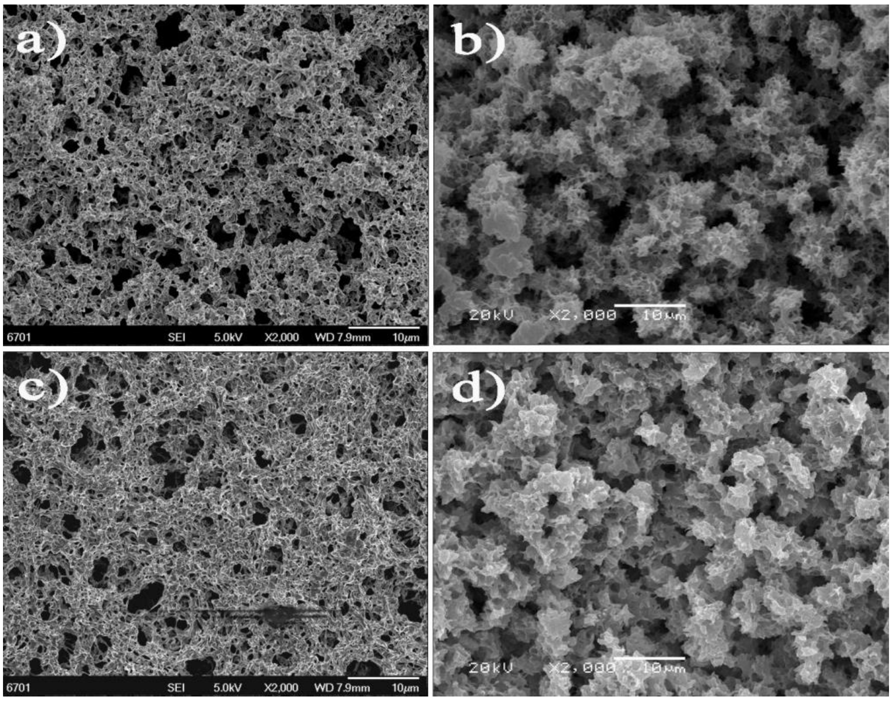

Figure S5. (a,b) surface and cross sectional structure images of the PVDF-F127-P (MMA-AA) membrane. (c,d) surface and cross sectional structure images of the PVDF-F127-P (MMA-AA) membrane after heating treatment.
